# Wireless Mid-Infrared Spectroscopy Sensor Network for Automatic Carbon Dioxide Fertilization in a Greenhouse Environment

**DOI:** 10.3390/s16111941

**Published:** 2016-11-18

**Authors:** Jianing Wang, Xintao Niu, Lingjiao Zheng, Chuantao Zheng, Yiding Wang

**Affiliations:** 1 State Key Laboratory on Integrated Optoelectronics, College of Electronic Science and Engineering, Jilin University, Changchun 130012, China; wjn14@mails.jlu.edu.cn (J.W.); niuxt15@mails.jlu.edu.cn (X.N.); zhenglj14@mails.jlu.edu.cn (L.Z.); 2 Electrical and Computer Engineering Department, Rice University, 6100 Main Street, Houston 77005, TX, USA

**Keywords:** non-dispersive infrared, CO_2_ sensor, wireless sensor networks (WSN), greenhouse

## Abstract

In this paper, a wireless mid-infrared spectroscopy sensor network was designed and implemented for carbon dioxide fertilization in a greenhouse environment. A mid-infrared carbon dioxide (CO_2_) sensor based on non-dispersive infrared (NDIR) with the functionalities of wireless communication and anti-condensation prevention was realized as the sensor node. Smart transmission power regulation was applied in the wireless sensor network, according to the Received Signal Strength Indication (RSSI), to realize high communication stability and low-power consumption deployment. Besides real-time monitoring, this system also provides a CO_2_ control facility for manual and automatic control through a LabVIEW platform. According to simulations and field tests, the implemented sensor node has a satisfying anti-condensation ability and reliable measurement performance on CO_2_ concentrations ranging from 30 ppm to 5000 ppm. As an application, based on the Fuzzy proportional, integral, and derivative (PID) algorithm realized on a LabVIEW platform, the CO_2_ concentration was regulated to some desired concentrations, such as 800 ppm and 1200 ppm, in 30 min with a controlled fluctuation of <±35 ppm in an acre of greenhouse.

## 1. Introduction

With the integration of agricultural and IT technology, accurate real-time monitoring and control systems for precision agriculture, like greenhouses, has been a desired requirement. Compared with traditional labor-intensive agriculture, reasonable environmental regulation, such as CO_2_ concentrations in the greenhouse, can effectively increase crop yield [1,2]. On the other hand, particularly in some developing countries, like China, with a relatively large demand in terms of grain storage and transport, the security of crops has become a significant issue. Therefore, a reasonable method to further reduce potential product losses caused by unpredictable harmful factors is of great importance in current agriculture. Therefore, vertical farming technology with accurate monitoring and control of agricultural factors has gradually attracted more attention in precise agriculture [3,4,5].

Besides the existing controllable environmental factors, including temperature and humidity, the potential effectiveness of CO_2_ fertilization in the greenhouse requires accurate and reliable detection and a smart control on CO_2_ concentration. Due to special application environmental conditions, the existing CO_2_ sensing techniques, including semiconductor, electrochemical, and infrared absorption spectroscopy [6,7], are greatly restricted and limited. For example, the mixed multi gas species in the greenhouse limit the feasibility of the semiconductor sensors. The commonly used electrochemical sensors, such as the commercially available MG811 from Winsen, have some inevitable shortcomings in sensing performances, including limited detection range, low accuracy, and high power consumption [7,8]. Among the detailed sensing technologies, like direct absorption spectroscopy (DAS) [9,10], photo-acoustic spectroscopy (PAS) [11], and tunable diode laser absorption spectroscopy (TDLAS) [12,13,14], because of its cost-effectiveness and feasibility in special applications in the environment of the greenhouse, the utilization of DAS sensing technology is reasonable and becoming increasingly popular.

As a communication tool between the control platform and sensor nodes, wireless sensor networks (WSNs) have been deployed as a reasonable and low-cost communication technology which can avoid the costly installment of telecommunication infrastructures and wired communications [15]. Compared with Power Line Communication (PLC), the WSNs have advantages including fault-tolerant ability, multi-faceted detection based on high redundant deployments, and fixable installment ability in complex environments. Besides the application in prevention and early detection of forest fires [16], an application of real-time monitoring and smart fertilization control has been significantly developed in intelligent agriculture [17,18], like smart compositing monitoring and control [19]. In addition, the relatively small size, implying a flexible integration with the sensor node, provides the possibility for managers to realize a unified management for an extensive planting area. In the application of wireless communication, some technologies, like Wi-Fi and Bluetooth, are mature and commonly used in the smart home [20]. However, due to the inherent data transmission rate characteristic which does not target the application for WSNs in the greenhouse, there is a relatively severe penalty in energy consumption. By contrast, wireless communication technologies at 443 MHz have a relatively strong penetration, indicating a long transmission distance for dealing with the inevitable plant shelter, which is a key advantage in its application in the greenhouse. Moreover, the integration between Global System for Mobile Communication (GSM) communication and WSN technology further enhances remote monitoring and control. In the current research, a Digital Enhanced Cordless Telecommunications (DECT) subnet is connected to a GSM coordinator [21]. In the actual application circumstance, the power consumption, node sides, and cost-effectiveness would be significant challenges, which would also restrict the selection and application in data transmission [22].

With the development of precision agriculture, traditional manual management and testing instruments were grandly replaced by the automatic developed smart terminal based on PC software like LabVIEW and LabWindows [23]. LabVIEW software from National Instruments (NI) Company is programmed by visual graphic G language. A development environment of LabVIEW creates flexible and scalable test and control applications. This software is able to make full use of computer resources to generalize instrument hardware, such as the expensive Data Acquisition (DAQ) board and related matching circuits. With the wide application range from basic interface to complicated signal processing, the sophisticated optimized program can be integrated into the background of the user interface to improve the compatibility and adaptation.

Since the first greenhouse was established in China in the late 1970s, the crop yield from solar greenhouses has increased by more than 3400 km^2^ from 1820 to 2010. With the further development of precision agriculture, this technique, which improves crop yield by artificially increasing CO_2_ concentration, has increased economic profits by ~30%, reported by the official assessment [1]. However, with the calibration of long-term monitoring of historical data of plants and utilization of trial and error, the optimized CO_2_ concentration in the growth of crops still extremely depends on experience. Besides the crop yield, the CO_2_ gas fertilization also has beneficial effects on the maturing period of crops and ability to resist diseases and pests. In sum, with a reliable monitoring system, automatic and accurate CO_2_ fertilization is desired in the development of the greenhouse.

What is more, this system regards accurate detection and control as the major purpose, and meanwhile tries to balance the benefit trade-off in reducing the engineering cost by optimizing measurement and control components. The smart WSN working mode based on RSSI (Received Signal Strength Indication) evaluation can adjust the transmission power automatically to increase the service life of the sensor nodes. In addition, the tolerable communication error rate can be ensured at the same time. With the basic support of monitoring and communication functions, the control facilities can realize a desired CO_2_ concentration regulation based on Fuzzy proportional, integral, and derivative (Fuzzy-PID) in the greenhouse.

The structure of this paper is organized as follows. In Section 2, the structure of this monitoring and control system is described, including a fabricated NDIR CO_2_ sensor working as the sensor node in WSN and related Fuzzy-PID control. In Section 3, a series of experiments in the laboratory are carried out to evaluate the system. The Fuzzy-PID regulation function is designed and implemented based on LabVIEW. In Section 4, a field deployment is performed to prove the desired functions, including environmental information monitoring, reliable wireless communication, and required CO_2_ concentration regulation. Finally, in Section 5, some conclusions are reached.

## 2. Structure of the Monitoring and Control System 

### 2.1. System Structure

**Figure 1:**
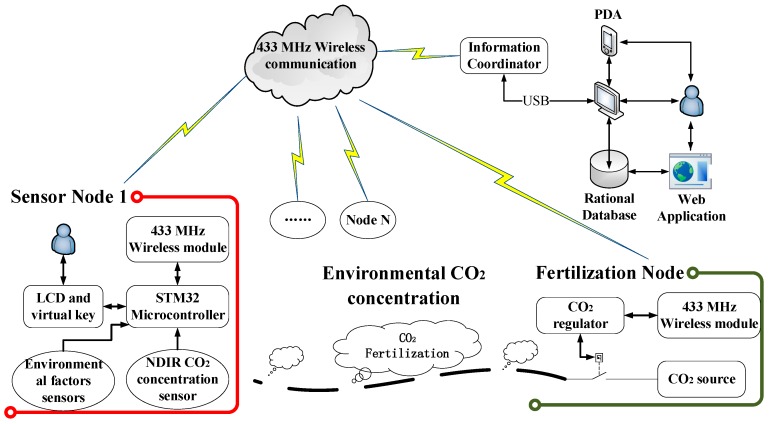
Structure of the CO_2_ monitoring and control system based on wireless network in a greenhouse environment.

The structure of this monitoring and control system is shown in Figure 1. There are four main sections. Firstly, the CO_2_ concentration sensor node based on NDIR technology with anti-condensation prevention and wireless communication is used to measure the required environmental factors and transmit data. Secondly, the established wireless sensor network realizes the function of data transmission and smart regulation of transmission power. Thirdly, the interactive software platform based on LabVIEW achieves signal processing and synchronization with the remote terminals. Finally, the control node fertilizes a suitable amount of CO_2_ in the greenhouse following the transmitted control parameters by the software platform on the terminal. These four sections establish a closed-loop monitoring and control system to realize CO_2_ measurement and fertilization.

### 2.2. Sensor Node Design and Implementation

#### 2.2.1. Infrared CO_2_ Sensor Design and Implementation

**Figure 2:**
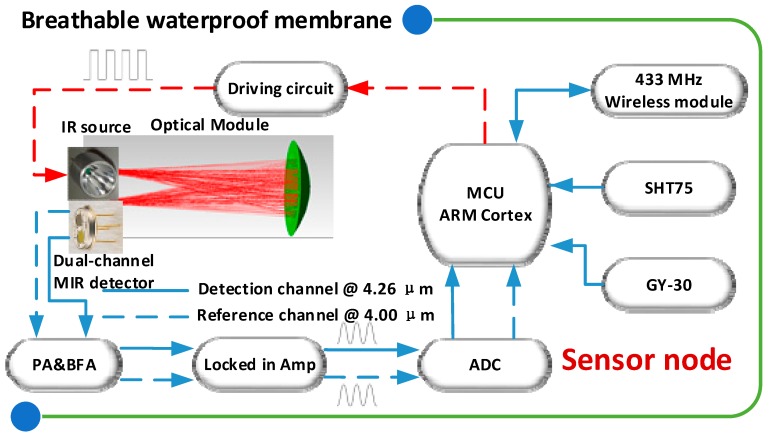
Structure of a CO_2_ sensor node using NDIR (non-dispersive infrared) technology.

The structure of a CO_2_ sensor node is shown in Figure 2. In order to ensure accurate CO_2_ fertilization, the CO_2_ sensor was designed and implemented based on infrared technologies with waterproof membranes prevention. The fabricated CO_2_ sensor includes two main parts, which are the optical part and electrical part. In the optical part, the used heat source (IR55, HawkEye, Milford, CT, USA) was fabricated with micro-electromechanical system (MEMS) technology and covering the required wavelength range. The pyroelectric detector (LIM262) (InfraTec, Dresden, Germany), equipped with two inherent filters, has a detectivity (D*) of 3.5 × 10^8^ $\mathrm{cm}\sqrt{\mathrm{Hz}}/W$ and receives the light reflected by a special spherical mirror, which was also used as a light-collector. In the electrical part, firstly, the IR heat source is driven by a desired signal. This signal was generated by micro-controller to MOSFET (Metal-Oxide-semiconductor Field-Effect Transistor). In consideration of it being fast switching and low on resistance, IRF830SPbF MOSFET was selected. Secondly, the generated signals by the detector are processed by a pre-amplifier (PA), a band-pass filter (BPF), a lock-in amplifier, and an analog-to-digital convertor (ADC). In the detection part, the pre-amplifier, whose core chip is INA116 (Texas Instruments, Dallas, TX, USA), plays a crucial role in suppressing noise and improving the signal-to-noise ratio (SNR). In the lock-in amplifier, the micro-controller was used to realize the digital phase shift function. The AD630 (Analog Device Inc., Norwood, MA, USA) works as the multiplier for the two-channel signals, with same frequency and phase. In the noise filter selection, an 8th-order Butterworth filter MAX291 (Maxim integrated, San Jose, CA, USA) was prioritized. In order to further improve the accuracy of digital–analog conversion, the low power consumption, 16-bit ADC MAX1416 (Maxim integrated, San Jose, CA, USA) was used to replace the internal 12-bit ADC of the micro-controller. This chip owns an inherent Σ-Δ with a digital filter. Finally, the measured data is packaged and sent to a 433-MHz wireless module. In the design and implementation of hardware, besides the basic function and performance, factors including energy consumption, scalability, and cost-effectiveness were significant reference indicators.

For measurement of environmental factors, a temperature and relative humidity multi sensor (type: SHT15, Sensirion, Laubisruetistrasse, Switzerland) was selected due to advantages including calibrated digital output, high reliability, and excellent long-term stability based on industrial CMOS processes with patented micro-machining (CMOSens^®^ technology, Laubisruetistrasse, Switzerland), which is characterized by the fusion of the sensor element and the signal conditioning electronics on a single silicon CMOS chip. Under this technology, a “micro-machined” finger electrode system with different protective and polymer cover layers forms the capacitance for the sensor chip, which protects the sensor from interference. A digital 16-bit ambient light sensor (type: BH1750FVI, ROHM, Tokyo, Japan) was selected for luminance measurement covering the desired ambient range from 400 nm to 710 nm, with a standby current low of up to 1.0 μA.

Before the light propagates to the detector, the light was filtered by two filter windows, corresponding to the detection channel at 4.26 µm and reference channel 4.00 µm. The detection channel and reference channel correspond to the strong absorption region of CO_2_ and nearly no absorption region of CO_2_, respectively. Because of the same background conditions, such as transmission path and light source, the environmental interference could be suppressed. In consideration of the modulation frequency limitation of both the IR heat source and the pyroelectric detector, the IR source was modulated by a 4 Hz square-wave signal. At both filter windows, the possible influential absorption interference from H_2_O and CH_4_ is five orders lower than CO_2_, so their interferences can be ignored. Since the two received optical beams by the dual-channel detector are highly related, a differential and ratio operation between them was performed to suppress the interferences from the sensor itself and possible outside interference in long-term utilization in the greenhouse.

The Beer–Lambert law in Equation (1) shows the relationship between the CO_2_ concentration and the received light intensity as:

(1) $$I\left(\lambda \right)=I_{0}\left(\lambda \right)\exp\left[-K\left(\lambda \right)CL\right]$$

where *I* is the light intensity received by the detector at λ, *I*_0_ is the initial emitting light intensity of the source at λ, *K* is the absorption coefficient, *C* is the CO_2_ concentration, and *L* is the optical length.

Under modulation, the time-dependent light intensities I(λ,t) at *λ*_1_ = 4.26 µm and *λ*_2_ = 4.00 µm will be converted to $u_{1}\left(\lambda_{1},t\right)$ and $u_{2}\left(\lambda_{2},t\right)$. Define their amplitude as *U*_1_ and *U*_2_, respectively. The final CO_2_ concentration could be expressed as a function of the differential voltage as:

(2) $$C=\frac{1}{KL}\ln[\frac{k_{1}}{k_{2}}(1-\frac{\Delta U}{U_{2}})]$$

In Equation (2), *k*_1_ and *k*_2_ are the relative optical-to-electrical conversion coefficients at *λ_1_* and *λ*_2_ respectively, ΔU is the difference between the amplitude of the detection signal (*U*_1_) and the amplitude of the reference signal (*U*_2_). Such a relation can be obtained through a calibration experiment. The actual relationship between the CO_2_ concentration and measured voltage signals can be achieved through calibration.

#### 2.2.2. Environmental Factors Sensing

A commercial SHT15 (Sensirion, Laubisruetistrasse, Switzerland), a relative humidity and temperature integrated sensor was selected. The accuracy of humidity and temperature are 3.0% and 0.4 °C, respectively. The best performance is near 28 °C and 80% humidity, which are applicable conditions in a greenhouse. Besides the inherent accuracy of the NDIR gas sensor itself, the calibration, depending on the application environment, is able to further enhance the performance of the CO_2_ sensor. In order to get more accurate environmental factors, the related calibrations are necessary. In addition, based on the working conditions, like voltage supply, temperature, humidity, and inherent ADC resolution, the nonlinear compensation of humidity detection and humidity compensation to temperature detection can be calibrated. Firstly, because of the inherent characteristic of the humidity sensor, the error from voltage dependence can be ignored. To compensate the non-linearity of the humidity result and obtain a full accuracy, the error from the ADC should be calculated and calibrated as:

(3) $$R_{\mathrm{Reading}}=c_{1}+c_{2}\times SO_{RH}+c_{3}\times SO_{RH}^{2}$$

where *SO_RH_* is a direct digital output from insider ADC, and *RH*_Reading_ is the calibration results. The linearization coefficients for humidity with the second-order polynomial fitting are decided according to the relationship between the standard relative humidity and the direct output result *SO_RH_* from the datasheet of RH and Temperature Non-Linearity Compensation. After the related fitting and calibration, the constant variables *c*_1_, *c*_2_, and *c*_3_ in Equation (3) match different values corresponding to the ADC sampling bits.

However, in the greenhouse, there is a large diurnal temperature, which indicates that the temperature coefficient of the relative humidity sensor should be considered as:

(4) $$RH_{\mathrm{Line}}=(T-25)\times (t_{1}+t_{2}\times SO_{RH})+RH_{\mathrm{Reading}}\;T=d_{1}+d_{2}\times T_{\mathrm{Reading}}$$

Equation (4) was used to calibrate the temperature result according to the power supply and ADC bits corresponding to the parameters *d*_1_, *d*_2_, and *T*_Reading_, respectively. Then the calibrated temperature was used to calibrate the errors caused by the temperature significant difference from 25 °C. In addition, *t*_1_ and *t*_2_ are two constant values depending on the ADC resolution.

BH1750FVI is a digital ambient light sensor a peak sensitivity wavelength of 560 nm and a high resolution from 1 to 65,535 lx. The measured luminance was converted by a 16-bit ADC and transmitted by I^2^C bus to the microcontroller. Because of the spectral responsivity of human eye, the measured ambient light can also be used to adjust the LCD power in the embedded terminal to save power.

#### 2.2.3. Waterproof Deployment for Anti-Condensation

The most serious constraint factor for the utilization of NDIR technology is the complicated environmental influence, such as the high humidity in the greenhouse. Even the DAS has a relatively wider application with better fault-tolerance; the dew condensation on the optical part will also bring severe errors. In order to satisfy the application requirement in the greenhouse environment, an intensively-designed waterproof breathable membrane was used as a protection layer for the chamber. Besides a compromise selection between waterproof ability and breathability, the abilities of anti-high-humidity and anti-high-temperature should also be considered. Therefore, the breathable waterproof membrane made of expanded Polytetrafluoroethylene (Eptfe) was selected and covered on the ventilation holes.

### 2.3. Wireless Communication

WSN communication protocol plays a fundamental role in data collection and feedback control. In the selection of a wireless module, low power consumption and stability which is specific as link budget, are two considerable factors. The core component selected in the wireless module is Si446x (Eptfe, Silicon Labs, Austin, TX, USA), including Si4460/61/63/64, with a working frequency range from 119 MHz to 1050 MHz and a link budget up to 146 dB. In consideration of cooperation with other research groups, Si4463 working in 433 MHz was the final option, with a maximum output power of 20 dBm (100 mW) and transmission current consumption of 70 mA. The optional multi-band switching makes the ad hoc networks possible in future development. Under the maximum transmission power of 100 mW, the corresponding ideal transmission distance is nearly 1000 m. However, the power should be adjusted reasonably according to the RSSI value. Although, the RSSI value, which implies the link quality, is vulnerable to the application environment, such as humidity, temperature and plant shelter conditions, and reliable transmission distance. Therefore, in consideration of a comprehensive influence in the greenhouse environment, link quality, co-channel interference, and plant shelter, the RSSI value was set in the range from −70 dBm to −90 dBm. For sensor nodes, the inherent transmission parameters of wireless modules, including configuration of the module channel, transmission power, and Identification (ID) number, can be adjusted by the micro controller.

**Figure 3:**
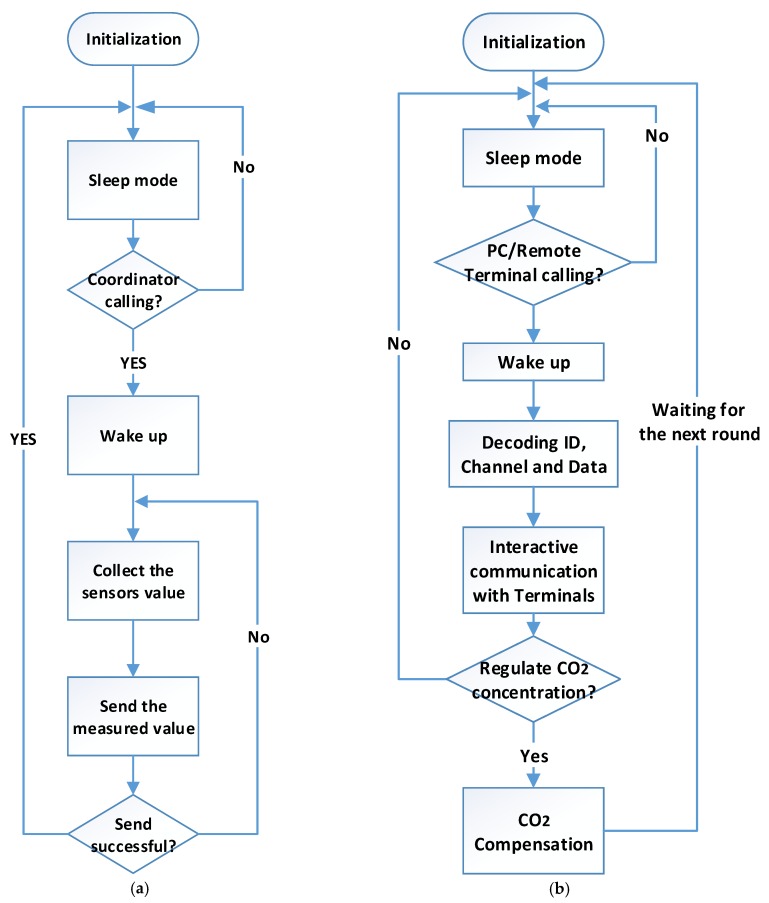
The flow charts of (**a**) communication process of sensor node; and (**b**) communication process of coordinator.

The detailed communication process is shown in Figure 3. In most cases, the sensor nodes are kept in listening mode, waiting for the call from the coordinator, to save power, except for special real-time monitoring required conditions. With a received command, such as data measurement and transmission power adjustment, a sensor node should complete it, and then sleep again, as shown in Figure 3a. However, the coordinator node does not collect the environmental information directly and only acts as a transmission part to coordinate the data transmission between the terminal PC and sensor nodes. The related control command is also transmitted through the coordinator node as shown in Figure 3b.

The data measured by sensor nodes were collected by the coordinator node as the input of the software platform. The control parameters determined by the related algorithm was transmitted to the controller facility to regulate the CO_2_ concentration. Then, the regulated CO_2_ concentration was measured by the sensor nodes again as the second round input. The interval of this closed-loop process was adjustable according to practical requirements. A short period indicates a relative timely feedback and accurate CO_2_ concentration regulation. As a comparison, a long period is able to decrease the power consumption. The trade-off between the control performance and power consumption should be taken into account according to large amounts of historical data and special requests of managers.

### 2.4. GUI Interface Based on LabVIEW

The interface platform was developed based on LabVIEW, which includes four main parts, including communication configuration, monitor, database, and dynamic Fuzzy-PID control. The communication configuration part realizes the configuration of the closed-loop bidirectional data transmission and the distribution of data cache. The received data and the recent variation trend will be displayed as the classified environmental factors in the monitor part. The link between a Structured Query Language (SQL) database and LabVIEW format is used to record the collected environmental information. The dynamic Fuzzy-PID controller is responsible for the whole operations, especially the CO_2_ concentration regulation. Besides the real-time CO_2_ concentration, the related environmental factors, such as luminance, temperature, and plant growth stage, are used to determine the dynamic Fuzzy-PID parameters for optimizing the regulation performance. In addition, the utilization of libraries in data-sharing realizes the function of remote monitoring and control. Both the browser and a Personal Digital Assistant (PDA) can act as remote terminals. Supported by GSM (core chip is SM900A, SIMCom, Shang Hai, China), the message is decoded as a mobile Attention (AT) instruction set and sent to a mobile for information broadcasting.

### 2.5. Fuzzy-PID Control

**Figure 4:**
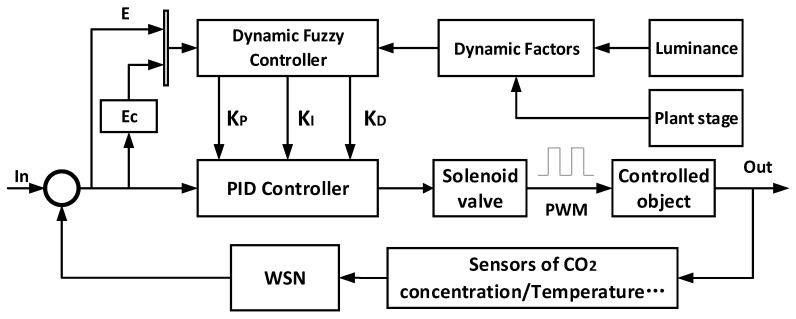
The diagram of the FLC (Fuzzy logic controller) for CO_2_ concentration regulation.

PID is a common method in engineering, and is the most widely used regulator control method and has a slight dependence on the accuracy of digital parameters. When the characteristics of the objects to be controlled are complicated and relatively non-linear or time-varying, the inappropriate parameter adjustment may cause oscillation in the system control. Compared with the traditional PID control, Fuzzy-PID controller has a relatively weak dependency on environmental factors, which is applicable in a complicated circumstance such as in a greenhouse. Based on the fuzzy logic principle [24], in most cases, a Fuzzy logic controller (FLC) shows a satisfying response capability for a non-linear system. The typical characteristic of an FLC is its incapability to generalize and only supply the response according to the existing rules. In this process, an expert in determining the inference logical rules can realize the function as a computation of desiring control value [25]. In practical application, in consideration of the relative data from the monitoring system for control purposes, an FLC is defined by a set of linguistic rules and fuzzy sets to compute the reasonable value for a greenhouse actuator. The actuator is the electromagnetic valve for relative regulation of CO_2_ concentration in this paper, as shown in Figure 4. The monitoring sensor nodes collect the required input factors and send them to the FLC. The FLC derive the required PWM driving signal for the valve to control the CO_2_ concentration. As the Fuzzy rules, a multiple inputs and multiple outputs (MIMO) are used in this system to regulate the CO_2_ concentration.

As shown in Figure 4, the multiple inputs come from the monitoring sensors, and the multiple outputs supply the required parameters for the following PID control. There are two inputs of the FLC, i.e., CO_2_ concentration error (*E*) and changing rate of error (*Ec*). The outputs of the FLC are the required parameters, *K*_P_, *K*_I_ and *K*_D_, for PID controllers. After the second calculation by the PID controller, the output value is set to the range from 1 to 100, which corresponds to the driving frequency of the actuator.

## 3. Laboratory Experiment and Measurement

### 3.1. Sensor Experiment

#### 3.1.1. Sensor Calibration

Standard gas samples preparation is crucial for gas detection, due to the fact that the accuracy of curve fitting and calibration depends on the uncertainty of the distributed gas samples. In the following experiment, compared with the static injection distribution, including operation error, a dynamic gas distribution using a mass flow meter was adopted. A 5000 ppm CO_2_ sample with 2% uncertainty and a 99.999% pure N_2_ were used as the gas sources to distribute desired gas samples.

**Figure 5:**
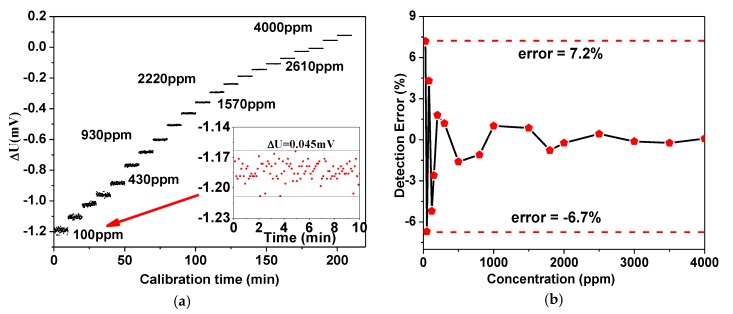
(**a**) Experimental data dots of the differential voltage (Δ*U*) versus the standard CO_2_ concentration. The inset shows the measured Δ*U* under 100 ppm for 10 min; (**b**) The measured detection errors for the prepared samples with the calibrated CO_2_ sensor.

In consideration of the actual circumstances in a greenhouse, the measurement calibration range was set to 0−4000 ppm. A series of gas samples distributed with a desired concentration was kept flushing the chamber and the data was recorded for >10 min until the chamber was completely filled with the gas sample, as shown in Figure 5a. According to the Lambert–Beer law, the relationship between the measured CO_2_ concentration (*C*) and the differential-ratio value between the two amplitudes of the output voltage signals (defined as *U*_1_ and *U*_2_) is

(5) $$C=43.43-1779.38\times \ln(\frac{0.2-\Delta U}{1.37})$$

Based on the measurement data shown in Figure 5a, the relative detection errors were calculated and plotted in Figure 5b. The maximum detection error occurs when the measured gas sample is near 0 ppm which is 7.2%, and the detection error became smaller with the increasing of the concentration.

#### 3.1.2. Limitation of Detection (LoD) Measurement

**Figure 6:**
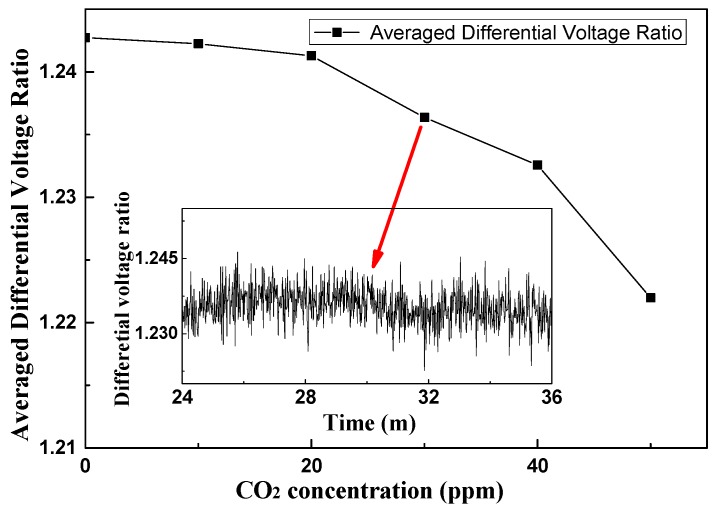
The measured averaged differential voltage (Δ*U*) versus the CO_2_ concentration within 0–50 ppm. The inset shows the recorded differential voltage (Δ*U*) results of a 30 ppm sample for 10 min.

In the LoD measurement, the chamber was flushed by a 99.999% N_2_ to avoid influence from residual gases. Then, the CO_2_ concentration was increased by 10 ppm. The measurement results are shown in Figure 6. Without any algorithms for optimization, compared with the nearly invisible voltage difference between 10 ppm to 20 ppm, there is a relatively much clearer voltage difference when the CO_2_ concentration was increased to 30 ppm. So, the LoD of the sensor was determined to be <30 ppm with a 0.8% fluctuation. It would be a rare case that the CO_2_ concentration was reduced to 100 ppm or less in a greenhouse. This indicates an acceptable measurement LoD and detection error of the sensor node for the application in such an environment.

#### 3.1.3. Stability

**Figure 7:**
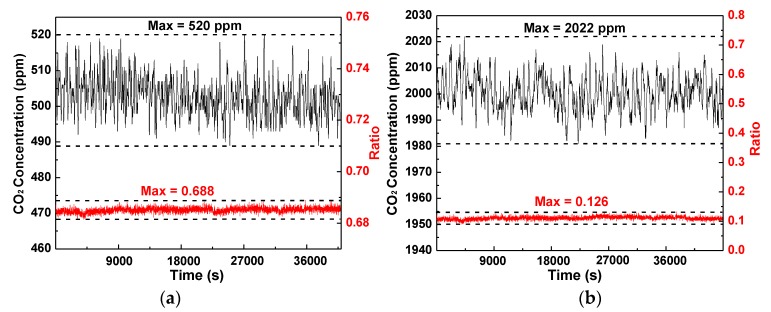
(**a**) Long-term measurement results on a CO_2_ standard sample with a concentration level of 500 ppm; (**b**) Long-term measurement results on a CO_2_ standard sample with a concentration level of 2000 ppm.

Two CO_2_ samples with concentration levels of 500 ppm and 2000 ppm were used corresponding to the normal CO_2_ concentration and suitable CO_2_ concentration for plant photosynthesis in a greenhouse. Based on the results shown in Figure 7a, the differential voltage was from 0.682 to 0.688. The peak differential voltage is 0.688, corresponding to a peak measured CO_2_ concentration of 520 ppm. Similarly, for the measurement of the 2000 ppm sample, the differential voltage was from 0.104 to 0.126, and the peak voltage and concentration were 1981 ppm and 2022 ppm, as shown in Figure 7b. In summary, considering the noises and interference, the fluctuations of 1.11% (22/2000) and 4% (20/500) of the measurement results on the 2000 ppm and 500 ppm samples, respectively, are acceptable in agriculture application.

**Figure 8:**
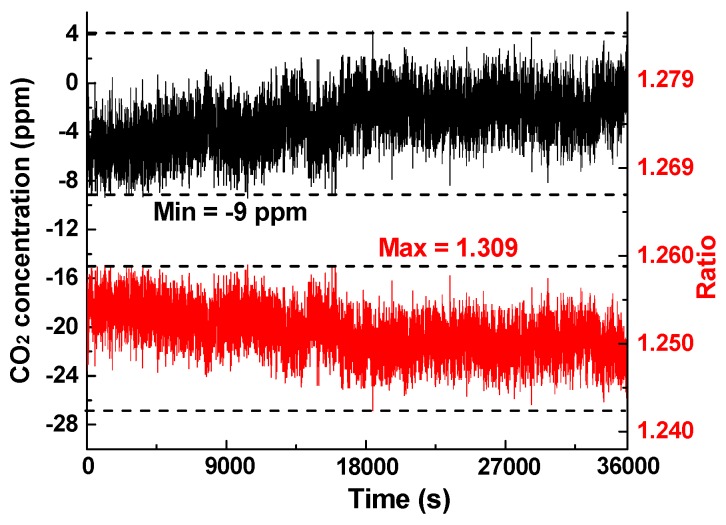
Long-term measurement results on a 0-ppm CO_2_ sample (pure N_2_) for 10 h with a 1-s sampling period.

Pure N_2_ was used to derive the theoretical LoD and the relationship between system noise and averaging time. The measurement results for 10 h are shown in Figure 8, including the differential voltage in the lower branch and the converted CO_2_ concentration in the upper branch using Equation (5). The peak fluctuation value of the converted concentration is −9 ppm, with an average of −2.8912 ppm, and the standard deviation was 2.2322 ppm.

**Figure 9:**
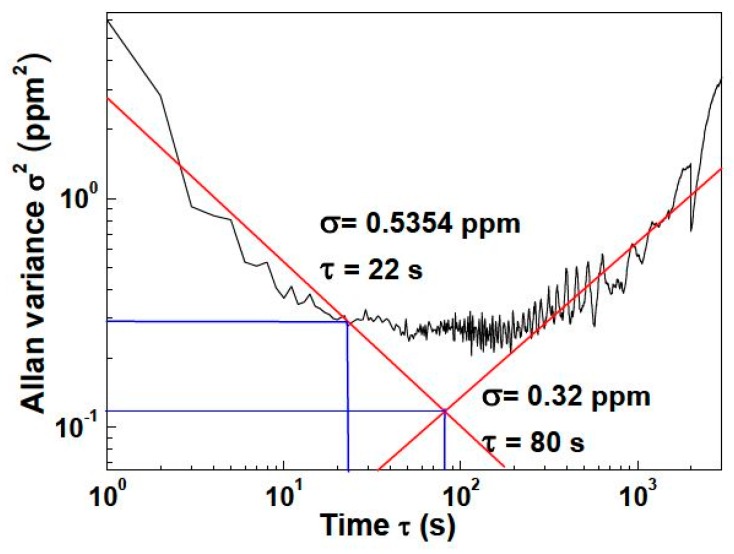
The Allan variance plot of this sensor at a pure N_2_ atmosphere.

According to the long-term experiment on the 0 ppm sample (pure N_2_), the Allan variance of this sensor was plotted in Figure 9. When the averaging time is 1 s, the theoretical detection limit is nearly 6 ppm. Based on the relationship between the noise influence and integration time, when the curve slope is nearly −1, the sensor was dominated by the white noise, and this situation ends at nearly 22 s integration time. Under this situation, the theoretical detection limit is 0.5354 ppm. In the intersection point of 1/2 slope, the Allan variance reaches the lowest value corresponding to the theoretical detection limit at the price of 80 s response time. The possible noise comes from temperature drift or certain environmental influences under this situation. In consideration of the special environment in a greenhouse, which is a large delay system, the not stringent measurement requirement in response time indicates that it is appropriate to increase the averaging time to enhance the stability of the sensor.

#### 3.1.4. Comparison among Some Commercial Sensors and the Sensor in This Work

The NDIR sensors in intelligent agriculture own some advantages compared with other kinds of sensors, such as semiconductor and electrochemical ones. As for CO_2_ sensors, some commercial NDIR sensors are designed and implemented by Senseair and ELT Sensor Companies. Compared with typical commercial sensors, the developed sensor has more satisfying precision and targets special application in solar greenhouses in the north of China in CO_2_ fertilization. Firstly, commercial sensors, such as K30 (Senseair Company) and S300 (ELT Sensor Company) [26], process the received data depending on the software algorithm to realize its miniaturization. The stability (3% of reading ±50 ppm) and LoD (nearly 50 ppm measured, even the measurement range in the datasheet is 0–5000 ppm), are insufficient compared with our fabricated NDIR sensor. Secondly, in our design, a two-channel pyroelectric detector was used to replace the commercial single-channel detector. After differential operation, environmental noises were suppressed, generating a high SNR and good long-term stability. Thirdly, both temperature and humidity sensors were integrated in the fabricated sensor node. The temperature of the monitored optical part was used to decrease temperature drift. Finally, in consideration of CO_2_ concentration control, a related higher sampling frequency can improve the control precision. However, the slow response time caused by difficult diffusion and slow modulation frequency of typical commercial detectors significantly limits the control precision. 

However, even the fabricated first-prototype sensor has a satisfying performance in the application in greenhouse with CO_2_ fertilization; a further miniaturization of sensor node size is expected that the current node size is 14 cm × 15 cm × 12 cm. Under 4 Hz modulation mode, the max average power consumption is nearly up to 0.98 W which is not acceptable to a self-powered sensor node. And the main power consumption comes from the light source driving. However, environmental information sampling ratio is adjustable according to the terminal commands for reasonable energy distribution. For example, at night, without CO_2_ fertilization, the sampling ratio could be set to a very low speed to save the power instead of the fixed sampling ration of typical commercial sensors. It ensured that a large capacity lithium battery is feasible to be used as a power supply.

### 3.2. Dynamic Fuzzy-PID Controller Based on the LabVIEW Platform

#### 3.2.1. Dynamic Fuzzy-PID Control Program

**Figure 10:**
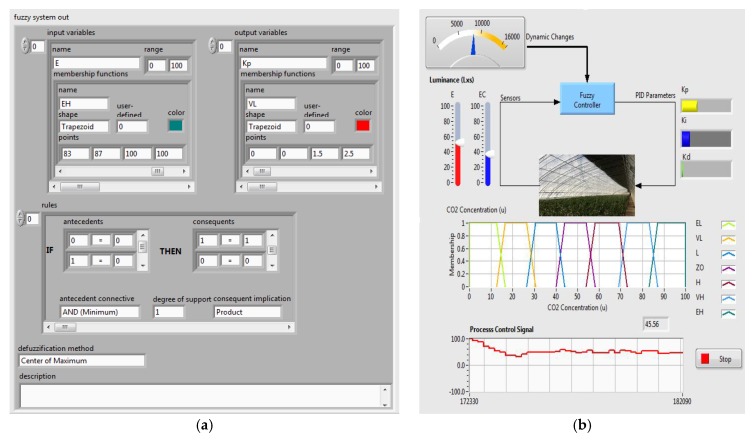
(**a**) Background dynamic Fuzzy-PID interface; (**b**) Interactive platform of the control module.

In the implementation of the dynamic Fuzzy-PID controller based on the LabVIEW platform, the desired functions of dynamic Fuzzy-PID algorithm and real-time monitoring and control were realized by a background program platform, shown in Figure 10a, and an interface platform, shown in Figure 10b.

The whole fuzzy calculation was performed by the background programs, including the parameters definition, fuzzy rules definition, and selection of the optional defuzzification methods. In the Fuzzy-PID algorithm, the input and output parameters are the real-time measured *E* and *Ec*, and PID parameters which were already normalized and distributed from 1 to 100. Another significant point in the same cluster with parameters definition is the membership set. Under normal circumstances, the membership functions sets and relative optimization depend on a test and error strategy from Math simulation. However, in order to adapt to environment and reach a good performance through careful tuning, the membership functions sets were decided by the combination between agricultural experience and specific characteristics in a greenhouse. The limitation of the greenhouse seal ability and the atmospheric CO_2_ concentration were also considered in the membership function. In addition, there would be an obvious concentration difference between inside and outside of the greenhouse when the photosynthesis is strong, caused by plenty of light. Under this situation, the diffusion phenomenon from high concentration to low concentration is dramatic. Therefore, the membership function can be adjusted online. After the set of the membership, the results were calculated following the fuzzy rules, which are extremely dependent on the experience and knowledge of experts and able to be obviously optimized by trial and error through several field experiments. Due to the seven classifications in membership, 49 rules were set to be “if…then…” format in the array. In the defuzzification methods set, the mode (center of the maximum), which is one of the common defuzzification options, was used. This is supported by the Fuzzy tools Library in LabVIEW. After the defuzzification process, the fuzzy values were converted to explicit control signals as the parameters of the following PID controller.

#### 3.2.2. Dynamic Fuzzy-PID Control Interface

According to the background program, the visible interface platform displays the changing real-time parameters and related control signals corresponding to the driving frequency of the solenoid valve. As an example, the adjustable seven linguistic variables were used (EL, extra low; VL, very low; L, low; ZO, zero; H, high; VH, very high; EH, extra high), which are shown by different colors matching the current difference. In this case, each possible linguistic value of inputs was assigned to an output linguistic value. Besides the normal Fuzzy-PID control process, certain influential factors in the applied circumstance should be considered. In a solar greenhouse, the most noteworthy variable is the time-varying luminance and plant growth stage, which significantly affect the intensity of photosynthesis. This has a direct relation to CO_2_ consumption and requirement. In addition, in this Fuzzy PID control system, the luminance value acts as the reference adjustment factor to membership functions. With a high luminance, indicating a dramatic CO_2_ concentration decreasing because of photosynthesis, the membership functions value would be regulated to a strong complementary of gas fertilization. This set is able to increase the robustness in relation to the changing environmental circumstances, such as weather.

#### 3.2.3. Hardware Design of the Control Facility

**Figure 11:**
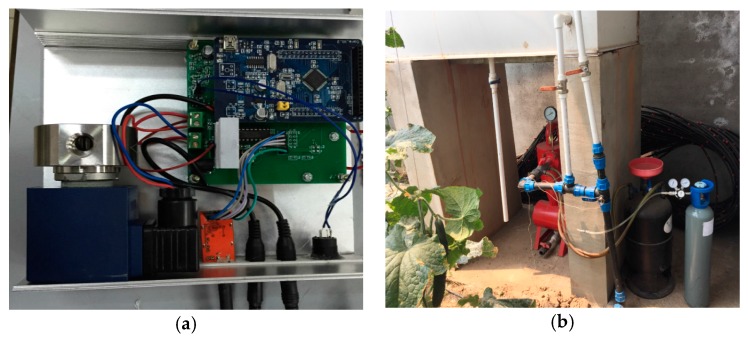
(**a**) The photo of the control facility; (**b**) The practical installment of control facilities in the greenhouse.

The control facility was an actuator which receives the control parameter and performs the actual electrical operation of the CO_2_ compensation in the greenhouse. The main components of the control facility included the wireless module, electric control part, electronic driving part, and solenoid valve, whose practical photo is shown in Figure 11a. A micro-controller processes and converts the received control parameters through the wireless module to a specific control signal for the 8-bit addressable latch (type: SN74LS259BN, Texas Instruments, Dallas, TX, USA), which enhanced the controllability and scalability. The electric signal indirectly controlled the electronic driving part through an optical coupler (type: TLP521, TOSHIBA, Tokyo, Japan) to isolate interference. In the electrical part, a logic control circuit composed of 8 Darlington (type: ULN2803, UTC, Shenzhen, China) supplied the required large driving current for the solenoid valve. A 99.99% CO_2_ gas in a 20 MPa cylinder was used as gas source for the compensation of CO_2_ concentration in the greenhouse. The outlet of the cylinder was connected to a pressure relief valve. The gas from the cylinder was delivered by a plastic catheter with diffusion holes spaced at a fixed distance, as shown in Figure 11b. Between the control facility and gas source, a pressure relief valve was used to reduce the pressure to less than 1 MPa. On cost principles, using the solenoid ZS-DN-5 (JVL, Shang Hai, China), whose power supply and control signal were transmitted by a coordinator node directly, less than 1-s lag was used for CO_2_ compensation.

## 4. Field Test Results and Discussion

### 4.1. Environmental Factors Measurement Results

**Figure 12:**
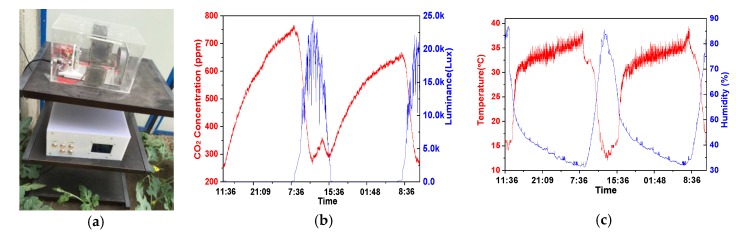
(**a**) The photo of a sensor node in a solar greenhouse; (**b**) The measured CO_2_ concentration and luminance in a greenhouse; (**c**) The measured temperature and humidity in a greenhouse.

In consideration of the application in a special environment like a greenhouse, some field tests was carried out to prove the ability and feasibility of this system. These tests were carried out in the Town of Shelin in Jilin Province, China. In field deployment, three nodes were fabricated and deployed in the greenhouse. The curve number in the software platform was able to monitor the information from different nodes. Through more than a one-week test, the stable and accurate performance with reasonable reproducibility indicated that the implemented system is applicable in a greenhouse environment. A practical installment photo is shown in Figure 12a, where the terminal monitoring and control software platform based on LabVIEW is embedded in an ARM A8 hardware platform in a white box. This sensor node is connected with the embedded terminal directly by a power line communication (PLC) because of a short distance. The interface is connected by the aviation plug. In other nodes, the data transmission line was replaced by a wireless transmission mode in WSNs to supply a fixable installment in the complicated environment of a greenhouse. In addition, wireless communication avoids the difficult wiring and corrosion to the lines in a PLC under complex circumstance in a greenhouse. However, in order to satisfy the driving power, the CO_2_ fertilization control facilities are controlled and driven by a PLC. Two-day measurement results, as an example, are shown in Figure 12b,c, including the CO_2_ concentration, luminance, temperature, and humidity. The repeated monitoring results were compliant with the photosynthesis law, which implies no detection errors during the two-day field test. From the measurement results, with plenty of light, the CO_2_ concentration rapidly decreased to 242 ppm, nearly 13:45, because of the strong plant photosynthesis. The lack of CO_2_ concentration suppressed the plant growth and decreased the crop yield. With the sunset, the luminance decreased to zero dynamically and the CO_2_ concentration began to accumulate because of the plant respiration. In addition, because the data was measured in April, the temperature is relatively high, up to 38 °C. In sum, through measurement results without human impact, the CO_2_ concentration was under a lacking state in the long-term. Therefore, a reasonable CO_2_ fertilization is a desired requirement to increase final production.

### 4.2. CO_2_ Concentration Regulation Results

**Figure 13:**
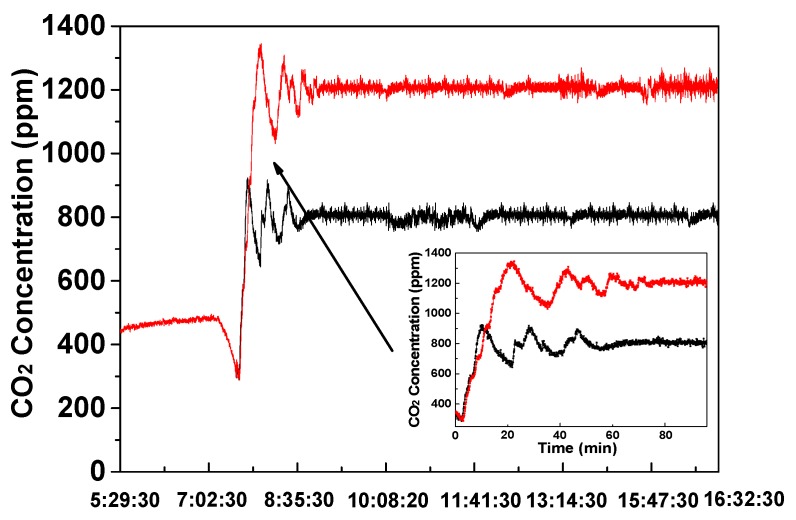
The measured CO_2_ concentration under dynamic Fuzzy-PID control.

In practical application, the operation frequency of the control facility is limited by the inherent 0.5 second delay of the solenoid valve. Therefore, the control parameters calculated by the FLC for adjusting the duty cycle and pulse width modulation of the electromagnetic valve was normalized to 1~10 from the original 1~100. In order to achieve the purpose of spreading evenly the compensated pure CO_2_ gas, the catheter through the whole greenhouse was opened, with a gas vent spacing at the same fixed distance. Even though, there was still an obvious diffusion delay corresponding to the overshoot and oscillation, shown in Figure 13. In the field test, the aimed CO_2_ concentration is 800 ppm and 1200 ppm, as examples responding to the different requirements of different plants. According to the test results, at least 40 min was required for the oscillation stage. In the 800 ppm control test, when the CO_2_ concentration under control was in the stable stage, the fluctuation range was from 762 ppm to 849 ppm, which was acceptable in the actual agricultural application. There was a similar result in the 1200 ppm control test. The CO_2_ concentration data in Figure 13 are the average results of the measurement result of each sensor node. In addition, some human-induced mutation was inevitable, because the actual field test environment was time-varying. However, a more reasonable membership and fuzzy rules are probably able to optimize the regulation performance.

### 4.3. RSSI Measurements

**Figure 14:**
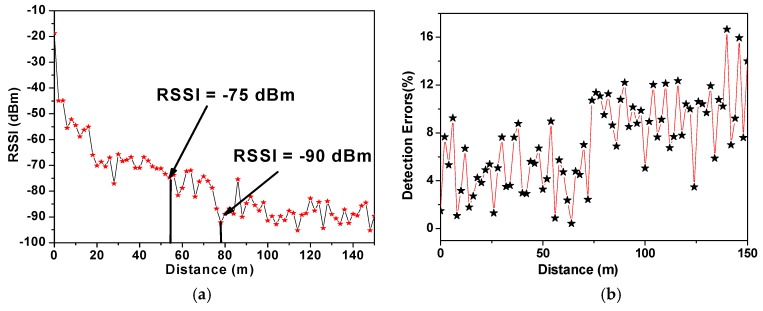
(**a**) The measured RSSI with increasing communication distance in a greenhouse; (**b**) the detection error of measured RSSI with the increasing communication distance in a greenhouse.

As the optional section of the radio transmission layer, RSSI is an indicator of the received signal strength to determine whether to increase the strength of the transmission power. Besides the transmission distance, which is the significant influential factor, the factors leading to changes of RSSI also include the directivity and gain of antenna, application environment, and the placement of the sensor node. In the field test, the CO_2_ sensor was placed in an 8 × 84 m^2^ watermelon greenhouse to carry out communication tests. The wireless communication module was powered by a standard 5 V voltage and the matched vertically-installed antenna was a common single whip type. Since the RF signal wavelength was affected by surface reflections, the sensor node was placed at half a meter from the ground, thus expecting to achieve the best communication performance. The initial transmission power was set to 20 dBm (100 mW). The field test results are shown in Figure 14. From Figure 14a, the relationship between the RSSI value and communication distance are basically consistent with the theoretical anti-exponential curve, with some fluctuation caused by the combined effect of regional plant shelter and inherent fluctuations. According to the preset feasible RSSI range, the corresponding reasonable transmission distance with 20 dBm transmission power is 58–80 m, which is able to satisfy the application in such a greenhouse. These test results were measured during the growth stage of the plants, and more serious signal attenuation in the harvest period from the relatively strong plant shelter influence should be paid more attention. Besides the transmission distance and plant shelter, the movement of mangers between the terminal PC and sensor nodes will bring nearly 10 dBm RSSI value attenuation, which is the tolerance range in the automatic transmission power regulation. In order to deal with the unpredictable influence on wireless communication from the environment, like humidity, temperature, and different plant shelter influences in different growth stages, the sensor nodes should intelligently adjust the transmission power to optimize the transmission performance, including saving power, ensuring link quality and avoiding co-channel interference.

However, there were undesired detection errors in the RSSI test and the detection errors increased with the transmission distance, as shown in Figure 14b. When the transmission distance is longer than 80 m, the detection errors reach 10%, implying that the method of using RSSI to evaluate link quality is not applicable in long-distance transmission in a greenhouse.

### 4.4. Data Sharing

**Figure 15:**
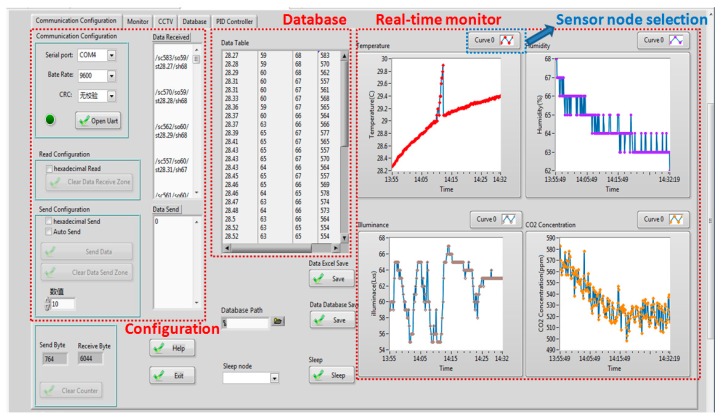
Data synchronization through a browser.

In order to satisfy remote monitoring and control requests, as seen in Figure 15, a GUI manager interface platform based on LabVIEW was developed as a web application server (WAS). The received packaged data were separated and displayed in the corresponding monitoring windows. From the blue dotted circle, the curve number corresponds to the sensor node number, which allows managers to monitor either the single node or multiple nodes. At the same time, the separated data were recorded in a database, Access 2007, linked and programmed by the Structured Query Language (SQL), which was also visible and shown in a data table in the front panel. In the communication process, when the received and sent bits recorded in real-time were over the deadline, a new database based on access would be established to avoid the continued occupancy of the local memory resources. Some optional configurations were supplied in the middle bottom of the plant, such as node sleep and database directions. Besides remote monitoring, there were also some related supports for remote control, which required Secure Sockets Layer (SLL) verification for network safety.

**Figure 16:**
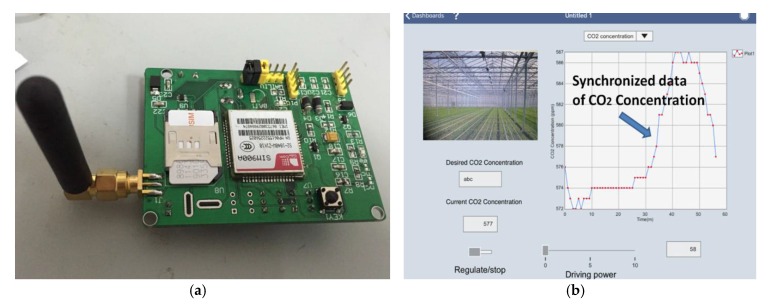
(**a**) Fabricated hardware circuit of the GSM communication; (**b**) Data synchronization through a Dashboard on an iPad.

In addition to web publishing, PDA terminals like IOS iPad and Android iPad are also able to realize remote monitoring and control functions based on a Dashboard, which is a terminal software application on LabVIEW, as shown in Figure 16b. The key point that should be paid attention to is the accurate matching between the virtual IP of the PC terminal and the actual IP of the mobile terminal, whose hardware circuit is shown in Figure 16a.

## 5. Conclusions

In this paper, a greenhouse monitoring and control system based on a differential mid-infrared CO_2_ concentration sensor and a dynamic Fuzzy-PID controller, respectively, was designed and implemented. With the moisture-proof structure, the fabricated sensor, based on NDIR technology, demonstrated a satisfying measurement performance, in the special range from 0 ppm to 4000 ppm corresponding to the common CO_2_ concentration in a greenhouse, and showed feasible application under high humidity conditions. The stability and reliability of communication via WSN was established, based on the RSSI measurement, to supply an adopted automatic adjustable transmission power at different stages of plant growth. The CO_2_ concentration in the greenhouse was controlled at a desired value, with a near 40-ppm fluctuation, according to the control parameters calculated by the dynamic Fuzzy-PID algorithm. Remote data monitoring and concentration control was also synchronized to the internet network through a browser and mobile terminals like iPad. The proposed system shows desired performance through field tests.
